# Biosynthesis of glycerol phosphate is associated with long-term potentiation in hippocampal neurons

**DOI:** 10.1007/s11306-016-1083-9

**Published:** 2016-07-23

**Authors:** Giuseppe Martano, Luca Murru, Edoardo Moretto, Laura Gerosa, Giulia Garrone, Vittorio Krogh, Maria Passafaro

**Affiliations:** 1 Institute of Neuroscience, CNR, Via L. Vanvitelli 32, 20129 Milan, Italy; 2 Fondazione IRCCS, Istituto Nazionale dei Tumori, Via Giacomo Venezian, 1, 20133 Milan, Italy

**Keywords:** Primary neurons, Glucose metabolism, Glycerol phosphate, Mass spectrometry, Long term potentiation, Hippocampus

## Abstract

**Introduction:**

Neurons have a very high energy requirement, and their metabolism is tightly regulated to ensure delivery of adequate substrate to sustain neuronal activity and neuroplastic changes. The mechanisms underlying the regulation of neuronal metabolism, however, are not completely clear.

**Objective:**

The objective of this study was to investigate the central carbon metabolism in neurons, in order to identify the regulatory pathways governing neuronal anabolism and catabolism.

**Methods:**

Here we first have applied MS-based endometabolomics to elucidate the metabolic dynamics in cultured hippocampal primary neurons. Using nanoLC-ESI-LTQ Orbitrap MS approach followed by statistical analysis, we measure the dynamics of uniformly labeled ^13^C-glucose entering neurons. We adapted the method by coupling offline patch-clamp setup with MS to confirm findings in vivo.

**Results:**

According to non-parametric statistical analysis of metabolic dynamics, in cultured hippocampal neurons, the glycerol phosphate shuttle is active and correlates with the metabolic flux in the pentose phosphate pathway. In the hippocampus, glycerol-3-phosphate biosynthesis was activated in response to long-term potentiation together with the upregulation of glycolysis and the TCA cycle, but was inactive or silenced in basal conditions.

**Conclusions:**

We identified the biosynthesis of glycerol-3-phosphate as a key regulator in mechanisms implicated in learning and memory. Notably, defects in enzymes linked with the glycerol phosphate shuttle have been implicated in neurological disorders and intellectual disability_._ These results could improve our understanding of the general mechanisms of learning and memory and facilitate the development of novel therapies for metabolic disorders linked with intellectual disability.

**Electronic supplementary material:**

The online version of this article (doi:10.1007/s11306-016-1083-9) contains supplementary material, which is available to authorized users.

## Introduction

Although the brain accounts for only 2 % of the total body mass (Mink et al. 1981), its energy consumption is high, accounting for about 20 % of the total energy expenditure and oxygen consumption. In areas and instances of increased brain activity, energy requirements are met by oxidative and non-oxidative metabolic activity and enhanced blood delivery (Ogawa et al. 1990; Attwell et al. 2010). High cellular energy needs are typically met by the upregulation of mitochondrial oxidation and increased flux through glycolysis. The main regulator of glycolysis is the cytosolic NAD/NADH ratio. Compared to other cell types, neurons possess peculiar features due to the presence of a metabolic coupling between astrocytes and neurons (Tsacopoulos and Magistretti 1996). Glucose is preferentially, although not exclusively, taken up by astrocytes (Chuquet et al. 2010; Jakoby et al. 2012), where it is processed and released as nutrients e.g. pyruvate, lactate, and glutamine, that could support neuron metabolism. Glycolysis in neurons is limited downstream of fructose-6-phosphate, regulated by the constant degradation of 6-phosphofructo-2-kinase/fructose-2, 6-bisphosphatase-3 (Herrero-Mendez et al. 2009). This glycolytic impediment promotes a flux in the direction of the pentose phosphate pathway (PPP). The oxidative phase of the PPP is critical for neuron survival because of the production of NADPH that is required to recycle glutathione, a compound that exists in low concentrations in neurons (Bolaños et al. 1996). On the other hand, non-oxidative PPP can provide glucose-6-phosphate and glyceraldehyde-3-phosphate (G3P) thus enabling the bypass of the glycolytic impediment. Furthermore, the astrocyte-to-neuron lactate-shuttle hypothesis (Pellerin and Magistretti 1994; Mächler et al. 2016) has resulted in differing theories on the mechanisms by which neurons keep glycolysis active (Chih and Roberts 2003; Mangia et al. 2011; Dienel 2012). When lactate is used as an energy substrate, the redox balance is maintained by NADH shuttle alone. Moreover, lactate dehydrogenation is governed by lactate dehydrogenase, which competes for NAD with glyceraldehyde 3-phosphate dehydrogenase; both these enzymes are highly expressed in neurons. There is more oxidized NAD in neurons than in astrocytes (Mongeon et al. 2016); therefore, both glycolysis and lactate uptake could be sustained. NAD regeneration appears to be very efficient in neurons, but the active mechanisms involved in the maintenance of NAD/NADH ratio remain unclear. Two pathways, the malate-aspartate shuttle and the glycerol phosphate shuttle, are the main regulators of this balance. The second pathway was considered inactive in the brain due to the lack of co-localization of the cytosolic and mitochondrial enzymes required for the shuttle (Nguyen et al. 2003). However, recent studies on the transcriptome in neurons showed that mRNA for both cytosolic and mitochondrial glycerol phosphate dehydrogenase are expressed in neurons (McKenna et al. 2006; Pardo and Contreras 2012). In this study, we investigated the central carbon metabolism in neurons, by using mass spectrometry (MS) techniques developed by Martano et al. (2015).

## Materials and methods

### Primary hippocampal neuron cultures

Primary hippocampal neuron cultures were prepared from Wistar rat embryos (Charles Rivers Laboratory Italia s.r.l.) euthanized at E18. Neurons were plated on coverslips in 12-well plates at a density of 75,000 cells/well. Preparation and seeding on glass coverslips were performed as previously described (Brewer et al. 1993).

For dynamic labelling experiments, isotopic switch was performed as described for adherent mammalian cells (Martano et al. 2015) by using a washing solution with 8.3 g/l DMEM (Sigma–Aldrich S.P.A., Italy) and 3.7 g/l NaHCO_3_. The medium for the isotope labelling switch had the same composition as the washing solution with ^U13C^glucose (Cambridge Isotope Laboratories Inc., USA.) 2.36 g/l added.

### Brain slice preparation and LTP induction

Two-month-old male C57BL6 mice were anesthetized with chloroform and euthanized. Experimental procedures were performed in accordance with the European Communities Council Directive (86/809/EEC) on the care and use of animals, and were approved by the Ethics Committees of the CNR Institute of Neuroscience in line with the ARRIVE guidelines (Kilkenny et al. 2010). Brain slices were prepared as described in literature (Folci et al. 2016) and transferred to oxygenated aCSF for 2 h prior to the experiments. aCSF contained 125.00 mM NaCl, 2.5 mM KCl, 1.25 mM NaH_2_PO_4_, 26 mM NaHCO_3_, and 25 mM glucose (all purchased from Sigma–Aldrich S.p.A., Italy). Compared with physiological cerebrospinal fluid, aCSF lack several carbon sources and required higher glucose concentrations than CSF. Physiological concentrations of glucose in aCSF are reported to cause depression of synaptic activity due to decreased glutamate release and decreased activation of α-amino-3-hydroxy-5-methyl-4-isoxazolepropionic acid receptor (AMPAR) that are necessary for LTP induction (Kamal et al. 1999). In order to minimize differences in the experimental conditions, slices for basal and LTP recordings were always prepared in parallel. Two slices at a time were transferred simultaneously into two different chambers and perfused with oxygen-saturated aCSF. Field excitatory post-synaptic potentials (fEPSPs) were recorded from the stratum radiatum of the hippocampal CA1 region after stimulation of the Schaffer collaterals, which were stimulated to evoke a half-maximal response. The activity in the slices was monitored in order to exclude samples that were critically damaged during preparation. After a baseline recording period of 10 min, LTP was induced by stimulating the Schaffer collateral pathway with one train of 100 stimuli at 250 Hz. The induction of LTP represented time point 0 of the experiments. For dynamic labelling experiments, the perfusion was performed with ^U13C^Gluc-aCSF containing 25 mM uniformly labelled ^13^C-glucose instead of natural glucose at the end of the baseline recording. Recordings were performed with a Multiclamp 700B amplifier (Axon CNS molecular devices, USA) and using an infrared-differential interference contrast microscope (Nikon FN-S2 N, Japan). Microelectrodes (borosilicate capillaries with a filament and an outer diameter of 1.5 µm, Sutter Instruments) were prepared with a four-step horizontal puller (Sutter Instruments) and had a resistance of 3–5 MΩ. Slices used for the evaluation of the pools were quenched after 60 min. Slices used for the evaluation of metabolic dynamics were quenched after 1, 5, and 60 min. Metabolism was quenched by cold shock in organic solvents and the hippocampus was rapidly dissected and collected for analysis.

### Sample preparation

Primary cultures on glass coverslip were fast-washed (approx. 1 s) in MilliQ water at 37 °C and immersed in a separate well containing acetonitrile, methanol, water, and formic acid (100:100:50:1) kept at −20 °C. Cells were detached using a cell scraper and samples were collected in a falcon tube (50 ml) and frozen with nitrogen.

Brain slices were transferred to a well with acetonitrile, methanol, and water (2:2:1) maintained at −20 °C to quench the metabolism. The hippocampus was rapidly dissected and transferred into a glass homogenizer containing 1 ml of acetonitrile, methanol, water, and formic acid (100:100:50:1), homogenized with a pestle, collected in a falcon tube, and frozen with nitrogen. Samples where lyophilized in a freeze-dry lyophilizer. The dry residue was reconstituted in water, sonicated, centrifuged (10,000 g, 3 min at 4 °C), and injected for LC–MS analysis.

### Analytical method

Chromatographic separation was performed using an Ultimate 3000 nanoLC system coupled with an Orbitrap Elite Hybrid Mass Spectrometer (Thermo Scientific, USA), and capillary column IF100-100H035 (NewObjective, USA). Mobile phases for analysis were (i) Mobile phase A: 450 µl H2O, 180 µl Tributylamine, 50 µl acidic acid buffered at pH 9.2 with ammonium hydroxide (ii) Mobile phase B: methanol. Separation was performed using a gradient from 90 % A to 10 % A in 15 min after injection and maintained with 10 % A for 10 min before re-equilibration with a constant flow of 0.5 µl/min. Injection was performed in low dispersion mode using 25 % of a 1 µl loop (250 nl). Mass acquisition was performed in negative mode with a resolution of 60000 and the inspected mass range was between 80 and 1100 m/z.

### Data mining, normalization, and statistics

Peaks were identified by matching the exact mass with the mass deviation below 3 part per million. Analyses were performed using eMZed2 software (Kiefer et al. 2013). Metabolites of interest were validated by standard addition with commercially available standards purchased from Sigma–Aldrich. Statistical analysis (i.e. *t* test and two-sample Kolmogorov–Smirnov test) were performed using the SciPy module in python (Oliphant et al. 2015). Cumulative concentrations of ATP, ADP, and AMP calculated from regression curves were used to normalize the data and calculate the fold change in the pool size. Compare with other normalization methods i.e. cell counting, this approach do not require to further extend the time for sample preparation prior quenching, thus avoid delays in the sample preparations which it may introduce unwanted metabolic perturbations. Moreover, it gives the possibility to correct analytical deviations, while normalization parameters obtained with parallel methods cannot (e.g. protein quantification).

## Results and discussion

### Central carbon metabolism in cultured neurons

**Fig. 1:**
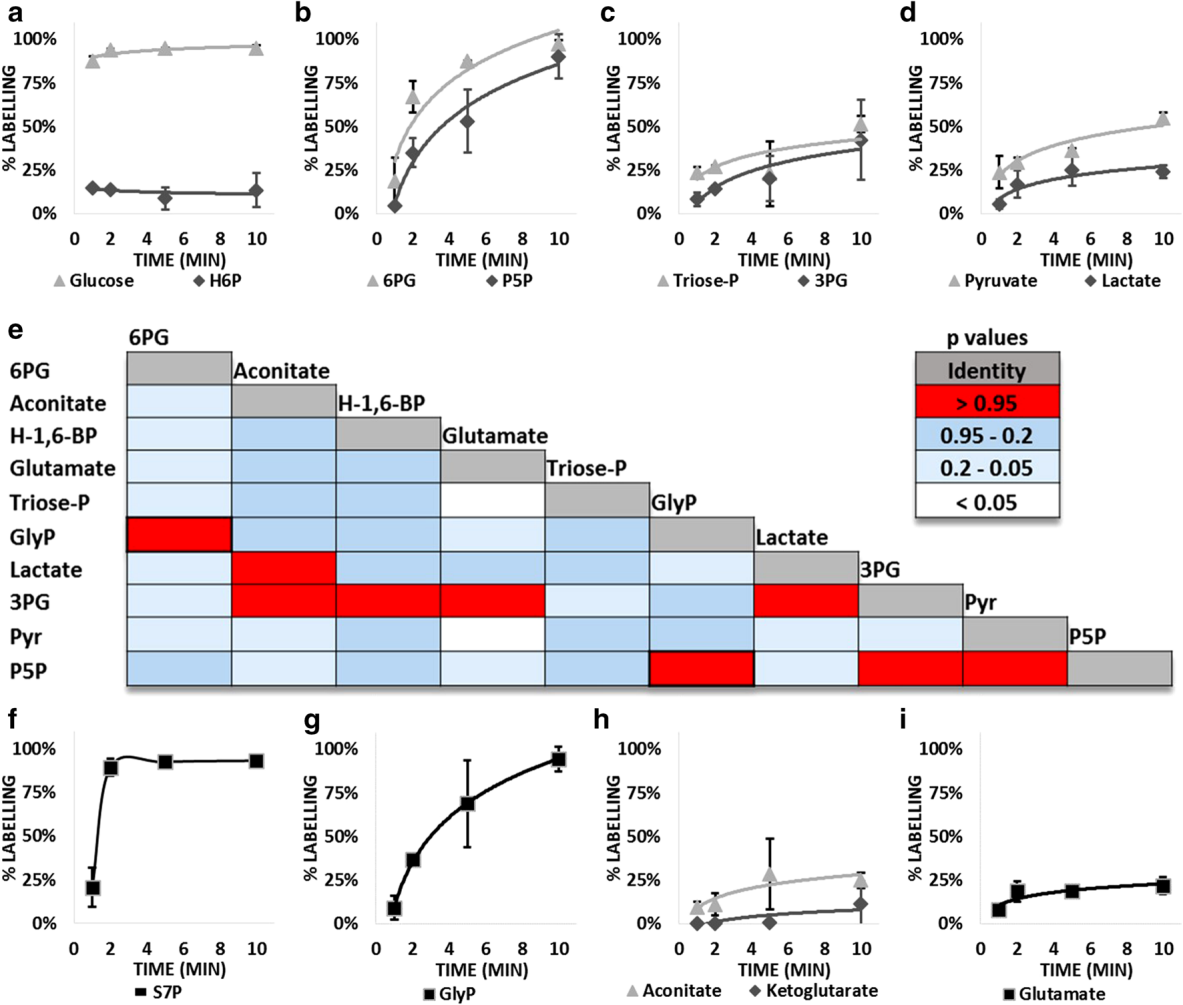
Dynamics and correlation of selected metabolites in the central carbon metabolism in hippocampal neurons. Switches were evaluated at 1, 2, 5, and 10 min with n = 4 for each point. **a** ^13^C dynamics of glucose and hexose-6-phosphate (H6P), **b** ^13^C dynamics of 6-phosphogluconate (6PG) and pentose-5-phosphate, (P5P), **c** ^13^C dynamics of triose-phosphate (Triose-P) and 3-phosphoglycerate (3PG), **d** ^13^C dynamics of pyruvate and lactate, **e**: graphical representation of p-values from two-sample Kolmogorov–Smirnov test. Metabolite dynamics where p > 0.95 or p < 0.05 were omitted. **f** ^13^C dynamics of sedoheptulose-7-phosphate (S7P), **g** ^13^C dynamics of glycerol-3-phosphate (GlyP), **h** ^13^C dynamics of aconitate and ketoglutarate, **i** ^13^C dynamics of glutamate

We studied the metabolism in neurons by determining fluxes in the central carbon metabolism. At 19th day in vitro (DIV19) hippocampal neurons were incubated for 1, 2, 5, and 10 min with uniformly labelled ^U−13C^glucose, and the label incorporation into intracellular metabolites was followed by MS. Intracellular glucose was nearly entirely exchanged with ^U−13C^glucose, reaching 87.9 % at minute 1 and more than 90 % at the other time points. Hexose-6-phosphates (H6P), comprising glucose-6-phosphate and fructose-6-phosphate, reached the steady state at the first inspected point (1 min), but with significant differences in the percentage of maximum labelling when compared to glucose (Fig. 1a; Table S1); a large portion of H6Ps appeared to be metabolically inactive or with a poor turnover. Downstream metabolites in the pentose phosphate pathway (PPP) (Fig. 1b; Table S1) and Embden-Meyerhof- Parnas (EMP) pathway (Fig. 1c and S1; Table S1) showed higher levels of labelling compared with H6Ps, indicating that approximately the 85 % of the H6P pool represents storage, which is not mobilized in these experimental conditions, thus limiting the maximum level of H6P labelling. Both oxidative (Fig. 1b) and non-oxidative phases (Fig. 1f; Table S1) of PPP display fast dynamism in neurons, with all the inspected metabolites showing more than 90 % of ^13^C after 10 min. The PPP oxidative phase is the most important source of reducing equivalent (i.e. NADPH), and produces ribose-5-phosphate, which serves as a precursor of nucleotide biosynthesis. The PPP is connected with the EMP-pathway via the non-oxidative phase governed by the enzymes transketolase and transaldolase. The rapid labelling of sedoheptulose-7-phosphate (S7P) and the formation of partially labelled isotopologues with three ^13^C in H6P, 6PG, and pentose-phosphate confirm the activity of the non-oxidative phase (Fig. S1). The increased percentage of labelling in the initial phase of analysis and the subsequent decrease are in agreement with the dynamics of triose-phosphate. Partially labelled intermediates are formed in the non-oxidative phase starting from S7P and G3P. Following the formation of pentose-5-phosphate with three labelled carbons, analysis showed a maximum concentration of this metabolite after 2 min and a subsequent decrease due to the ^13^C entering the precursor G3P. Notably, S7P presents a dynamic that is faster compared to that of precursors. Cultured neurons also have a small portion (<10 %) of cocultured astrocytes, which are required for proper development. Another issue that may influence these dynamics is the presence of other possible segregated pathways inside neurons, which are linked to the trafficking of mitochondria. Mitochondrial trafficking is very organized and responds rapidly to stimuli such as calcium influx regulated by neurotransmitters (Schwarz 2013; Beckervordersandforth et al. 2015). This creates metabolic niches, which may be responsible for unexpected dynamics. We measured the labelling entering glycerol-3-phosphate and observed high activity, with 94.5 % of glycerol-3-phosphate labelled after 10 min. Together with malate-aspartate shuttle, glycerol phosphate biosynthesis contributes to the balance of cytosolic NAD/NADH levels. Glycerol phosphate (GlyP) is connected with the EMP-pathway via the triose-phosphates. G3P is converted to dihydroxyacetone phosphate (DHAP) by triose-phosphate isomerase. The conversion of DHAP to GlyP is regulated by NADH-dependent cytoplasmic glycerol-3-phosphate dehydrogenase. Comparison of the regressions between the different metabolites by using the non-parametric two-sample Kolmogorov–Smirnov test (Fig. 1e), showed that GlyP correlates with the regression of 6PG and P5P (p > 0.95). Recent scrutiny of PPP has shown the importance of this mechanism in order to circumvent the metabolic bottleneck of glycolysis in neurons due to the limited ability to synthetize fructose-bisphosphate (Brekke et al. 2012; Bouzier-Sore and Bolaños 2015). PPP allows the simultaneous production of reducing equivalent and triose-phosphates that can fuel glycolysis. In contrast with cortical neurons, where the glucose entering the PPP was not recycled in glycolysis (Bolaños et al. 2010), we observed that downstream metabolites in the EMP-pathway, such as 3PG and pyruvate, correlated with P5P dynamics. Surprisingly, the dynamics of GlyP reflected the dynamics in the pentose phosphate pathway, but not those of the precursor triose-P. Thus, the biosynthesis of triose-P (i.e. G3P and DHAP) occurred from the EMP-pathway and PPP, but the triose-P entering the glycerol phosphate shuttle is derived from the PPP. Analysis of the TCA cycle and linked metabolites such as glutamate showed moderate labelling in the metabolites (Fig. 1h, i).

### Central carbon metabolism in the hippocampus in brain slices

**Fig. 2:**
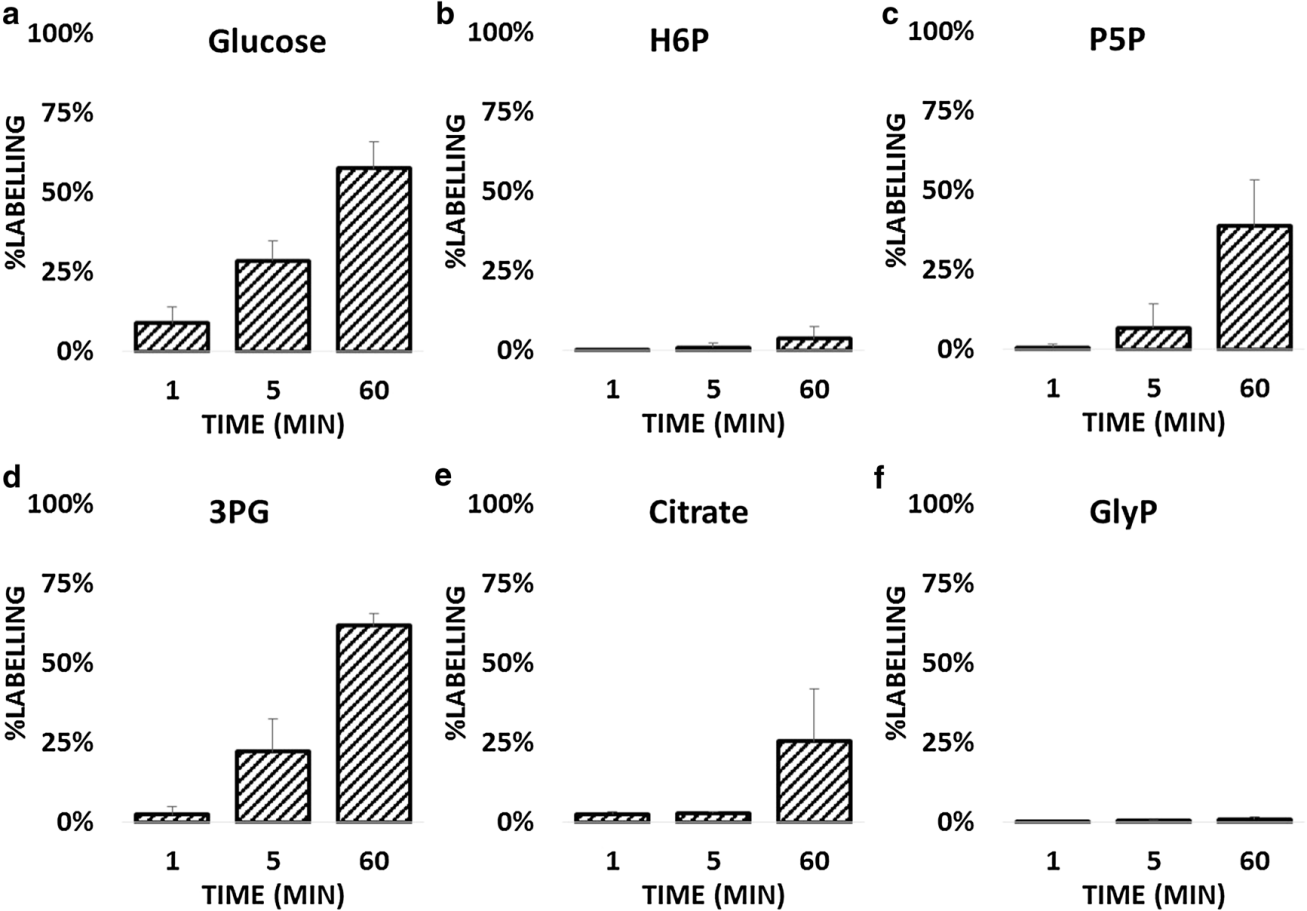
Dynamics of selected metabolites of central carbon metabolism in the hippocampal region from mouse brain slices. Switches were evaluated after 1, 5, and 60 min with n = 3 for each point **a** ^13^C dynamics of glucose, **b** ^13^C dynamics of hexose-6-phosphate (H6P), **c** ^13^C dynamics of pentose-5-phosphate (P5P), **d** ^13^C dynamics of 3-phosphoglycerate (3PG), **e** ^13^C dynamics of citrate, **f** ^13^C dynamics of glycerol-3-phosphate (GlyP)

Brain slices are more representative of physiological conditions than are primary cultured neurons, because of the presence of a fully developed network where glial cells outnumber neurons, and preservation of metabolic storage. Compared to neurons in culture, the time taken to load glucose into the cells was higher in slices (Fig. 2a; Table S2). Beside the time required for glucose to homogeneously distribute within the tissue, the neurons in the slices could also have a relatively lower dependency on glucose than in vitro cultures do, due to the availability of different stored nutrients in the tissue (i.e. glycogen in astrocytes, fatty acids, and residual nutrients in the circulatory system). H6P analysis (Fig. 2b; Table S2) showed poor labelling of these metabolites, only 3.6 % after 1 hour, confirming previous findings that the majority of the H6P pool is inactive in brain slices. Further similarity between in vitro and ex vivo conditions were observed in the dynamics of P5P (Fig. 2c), downstream glycolytic intermediate 3PG (Fig. 2d; Table S2), and the TCA cycle (Fig. 2e; Table S2). In contrast, the biosynthesis of glycerol-3-phosphate was not active (Fig. 2f; Table S2). This difference between cultured neurons and brain slices suggests that the role of glycerol-3-phosphate shuttle and the relative activation of this path may be environment-dependent. Cultured neurons lack connections with astrocytes, and the substrate availability is limited compared to that in brain slices. Therefore the observations could be an artefact specific to the in vitro environment. We conclude that this path is not required under basal conditions, probably because other pathways such as malate-aspartate shuttle and mitochondrial oxidative phosphorylation are sufficient to provide both ATP and NAD/NADH balance in the cytosol without requiring the activation of the glycerol phosphate shuttle. However, the ability to activate this pathway in a very efficient manner in cultured neurons suggests that this pathway could be required when the energy demand increases.

### Central carbon metabolism in response to long-term potentiation in brain slices

**Fig. 3:**
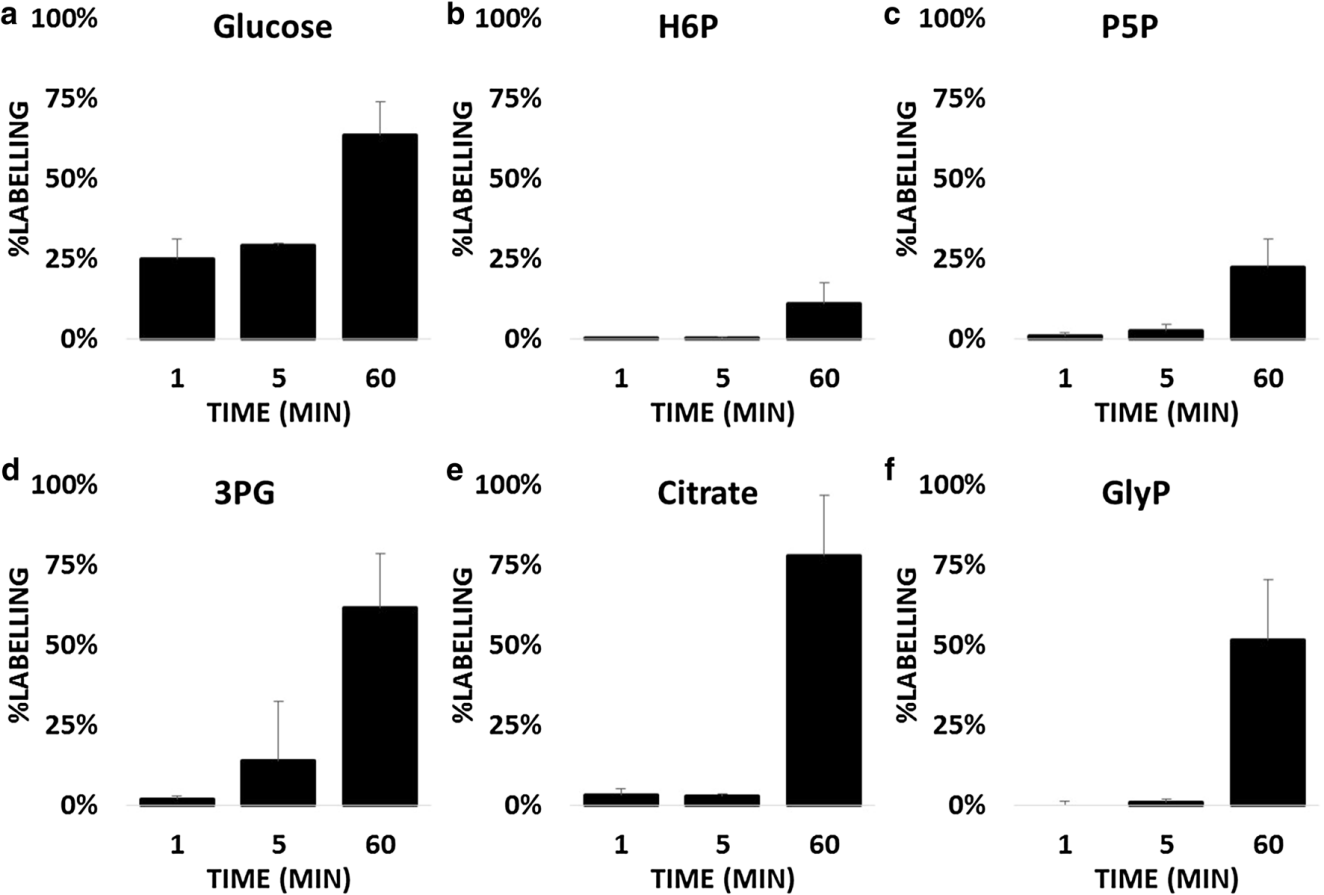
Dynamics of selected metabolites of central carbon metabolism in the hippocampal region from mouse brain slices after LTP induction. Switches were evaluated after 1, 5, and 60 min with n = 3 for each point **a** ^13^C dynamics of glucose, **b** ^13^C dynamics of hexose-6-phosphate (H6P), **c** ^13^C dynamics of pentose-5-phosphate (P5P), **d** ^13^C dynamics of 3-phosphoglycerate (3PG), **e** ^13^C dynamics of Citrate, **f** ^13^C dynamics of glycerol-3-phosphate (GlyP)

To determine whether the glycerol phosphate shuttle is required when energy requirements increase, we investigated the dynamics and changes in metabolic pools after the induction of long-term potentiation (LTP). LTP is considered the major path that underlies learning and memory (Bliss and Collingridge 1993). Maintenance of increased synaptic potentials, rather than action potentials, represents the main energy cost related to the maintenance of excitability (Bélanger et al. 2011). Therefore, it is expected that metabolism will be rerouted in order to sustain such prolonged energy-requiring activity. We compared metabolite concentrations after LTP and those under basal conditions and found no significant differences (Fig. S2). However, major differences were observed in the metabolic fluxes in response to LTP. In the first minute, the uptake rate of glucose (Fig. 3a; Table S3) increased significantly compared with basal conditions (p = 0.023); the difference was not significant at later time points (5 and 60 min). No statistically significant changes were observed downstream of glucose compared with basal conditions in the EMP pathway and PPP (Fig. 3b–d). As expected, the increased energy demand accelerates the dynamics in mitochondria, as shown by the citrate ^13^C loading at min 60 (Fig. 3e).The difference in citrate dynamics between the LTP and basal conditions was significant (p = 0.040). In line with our latest hypothesis, the biosynthesis of glycerol phosphate (Fig. 3f) was activated (p < 0.001) and reached approximately 50 % of the entire pool after LTP. Both astrocytes and neurons are capable of activating this pathway, although our observations may be unique to neurons. As previously mentioned, astrocytes can use lactate biosynthesis for increased glycolysis, thus balancing the cytosolic NAD/NADH and therefore might not need to activate glycerol phosphate shuttle. Other statistically significant differences were observed in the labelling in aspartate and glutamine (Fig. S3) biosynthesis. Both increase in response to LTP, from 2 to 6 % for aspartate and from 1 to 4 % for glutamine, whereas the majority of the pool was unlabeled after 1 h.

## Conclusions

**Fig. 4:**
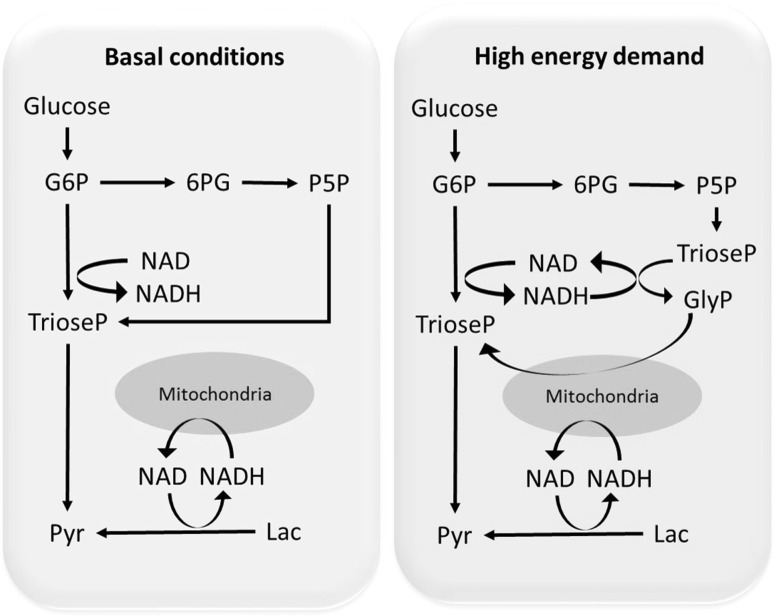
Schematic representation of glucose metabolism in neurons under basal conditions (*blue*) and in response to increased energy demand evoked by long-term potentiation (*pink*)

In this work, we used liquid chromatography coupled with mass spectrometry to measure the dynamic changes in the central carbon metabolism in hippocampal neurons. We found that GlyP biosynthesis is active in cultured neurons when glucose is the sole carbon source. GlyP biosynthesis is activated in the hippocampus when the energy demand increases in response to LTP. Moreover, the dynamic of the glycerol phosphate shuttle appears to be regulated by the triose-phosphates produced by PPP but not by those produced in the EMP pathway (Fig. 4). The activation of glycerol phosphate shuttle is likely essential to keep the NAD/NADH balance in the cytosol and therefore sustains both glycolysis and lactate conversion into pyruvate while providing ATP without downregulating the PPP and the production of reducing equivalents. Compared to other pathways (e.g. malate shuttle), glycerol phosphate shuttle is less efficient in energy production and is linked to increased release of reactive oxygen species (Mráček et al. 2013). This could be why this pathway is only activated when required, i.e. when the malate shuttle is insufficient to maintain the NAD balance. Defects in enzymes linked with this shuttle such as triose phosphate isomerase and mitochondrial glycerol phosphate dehydrogenase are implicated in neurological disorders (Orosz et al. 2009) and intellectual disability (Daoud et al. 2009). Thus, our findings contribute to our understanding of the general mechanisms of learning and memory and could facilitate the development of novel therapies for related metabolic disorders. Further studies on the modulation of the glycerol phosphate shuttle by using pharmacological approaches are also required to assess the risks and benefits of modulating this pathway, particularly to clarify the risks of glycerol phosphate shuttle modulator-based therapeutic agents such as metformin (Madiraju et al. 2014), a drug that can cross the blood brain barrier (Moreira 2014, Check 2015).

## Electronic supplementary material

Below is the link to the electronic supplementary material.

Supplementary material 1 (DOCX 105 kb)
