# Supplementary material for: Biosynthesis of glycerol phosphate is associated with long-term potentiation in hippocampal neurons
Source: Metabolomics. 2016 Jul 23;12:133. doi: 10.1007/s11306-016-1083-9 (PMC4958395; doi:10.1007/s11306-016-1083-9)
Supplement: Supplementary file 1 — Supplementary material 1 (DOCX 105 kb) [file 11306_2016_1083_MOESM1_ESM.docx]

**Supplementary Material**

| **EMP - Pathway** | 1 min | | | 2 min | | | 5 min | | | 10 min | | |
| --- | --- | --- | --- | --- | --- | --- | --- | --- | --- | --- | --- | --- |
| Glucose | 87.9% | ± | 2.4% | 93.8% | ± | 1.0% | 95.0% | ± | 0.2% | 94.9% | ± | 1.8% |
| Hexose-6-phosphate | 15.0% | ± | 1.5% | 14.0% | ± | 0.4% | 8.8% | ± | 6.5% | 13.6% | ± | 9.9% |
| Hexose-1,6-bisphosphate | 9.9% | ± | 10.7% | 6.4% | ± | 5.8% | 30.1% | ± | 17.8% | 55.0% | ± | 27.7% |
| Triose-phosphate | 23.6% | ± | 3.3% | 27.3% | ± | 0.5% | 23.0% | ± | 18.6% | 51.6% | ± | 4.7% |
| Phospho-D-glycerate | 8.3% | ± | 3.9% | 14.4% | ± | 1.7% | 20.3% | ± | 13.1% | 42.4% | ± | 23.0% |
| Pyruvate | 23.8% | ± | 9.4% | 29.7% | ± | 2.6% | 36.5% | ± | 1.3% | 54.9% | ± | 3.1% |
| **Pentose phosphate pathway** |  |  |  |  |  |  |  |  |  |  |  |  |
| 6-Phosphogluconate | 18.8% | ± | 13.4% | 67.0% | ± | 9.0% | 87.6% | ± | 0.5% | 97.5% | ± | 2.2% |
| Pentose-5-phosphate | 4.6% | ± | 1.0% | 35.1% | ± | 8.4% | 53.2% | ± | 18.2% | 90.3% | ± | 12.9% |
| Sedoheptulose 7-phosphate | 20.6% | ± | 11.3% | 89.4% | ± | 4.9% | 92.6% | ± | 3.3% | 93.3% | ± | 3.4% |
| **TCA Cycle** |  |  |  |  |  |  |  |  |  |  |  |  |
| Aconitate | 9.3% | ± | 3.3% | 11.2% | ± | 6.3% | 28.6% | ± | 20.2% | 24.9% | ± | 4.4% |
| Citrate | 0.2% | ± | 0.1% | 0.9% | ± | 0.6% | 3.6% | ± | 5.0% | 7.6% | ± | 5.5% |
| Ketoglutarate | 0.2% | ± | 0.1% | 0.2% | ± | 0.1% | 0.5% | ± | 0.2% | 11.5% | ± | 12.8% |
| Fumarate | 0.1% | ± | 0.1% | 0.1% | ± | 0.0% | 0.1% | ± | 0.0% | 0.1% | ± | 0.0% |
| Malate | 0.0% | ± | 0.0% | 0.0% | ± | 0.0% | 0.0% | ± | 0.0% | 0.5% | ± | 0.4% |
| Oxalosuccinate | 10.6% | ± | 6.7% | 21.3% | ± | 8.0% | 32.8% | ± | 20.4% | 28.9% | ± | 19.9% |
| **Other** |  |  |  |  |  |  |  |  |  |  |  |  |
| Glutamate | 7.7% | ± | 0.8% | 18.5% | ± | 6.0% | 18.9% | ± | 3.6% | 21.8% | ± | 4.8% |
| Glycerol 3-phosphate | 9.1% | ± | 6.9% | 36.7% | ± | 3.5% | 68.8% | ± | 24.7% | 94.5% | ± | 7.1% |
| Lactate | 5.6% | ± | 2.6% | 17.0% | ± | 7.7% | 25.0% | ± | 9.0% | 24.0% | ± | 3.7% |

Table S1: ^13^C dynamic labelling in *in vitro*-enriched hippocampal neurons from Wistar rat embryos at Div 19. n = 4 for each time point.

| **EMP - Pathway** | 1m | | | 5m | | | 60m | | |
| --- | --- | --- | --- | --- | --- | --- | --- | --- | --- |
| Glucose | 8.9% | ± | 5.1% | 28.5% | ± | 6.3% | 57.6% | ± | 8.6% |
| Hexose-6-Phosphate | 0.0% | ± | 0.0% | 0.9% | ± | 1.3% | 3.6% | ± | 4.0% |
| Hexose-1,6-Phosphate | 0.7% | ± | 0.7% | 0.1% | ± | 0.0% | 14.3% | ± | 5.3% |
| Triose-phosphate | 0.0% | ± | 0.0% | 0.2% | ± | 0.0% | 8.1% | ± | 6.0% |
| Phospho-D-glycerate | 2.3% | ± | 2.6% | 22.1% | ± | 10.3% | 61.8% | ± | 3.8% |
| **Pentose phosphate pathway** |  |  |  |  |  |  |  |  |  |
| Ribose-5-P | 0.6% | ± | 1.0% | 6.8% | ± | 7.5% | 38.8% | ± | 14.6% |
| **TCA Cycle** |  |  |  |  |  |  |  |  |  |
| 2-Ketoglutaric acid | 0.1% | ± | 0.0% | 0.0% | ± | 0.0% | 0.1% | ± | 0.0% |
| Aconitic acid | 0.1% | ± | 0.1% | 0.1% | ± | 0.1% | 0.1% | ± | 0.1% |
| Citric acid | 2.6% | ± | 0.6% | 2.9% | ± | 0.5% | 25.5% | ± | 16.5% |
| Succinic acid | 0.0% | ± | 0.0% | 0.0% | ± | 0.0% | 0.1% | ± | 0.1% |
| **Other** |  |  |  |  |  |  |  |  |  |
| L-Glutamate | 0.0% | ± | 0.0% | 0.3% | ± | 0.0% | 12.2% | ± | 2.5% |
| D-Aspartic acid | 0.0% | ± | 0.0% | 0.0% | ± | 0.0% | 2.5% | ± | 0.4% |
| GABA | 0.0% | ± | 0.0% | 0.0% | ± | 0.0% | 0.1% | ± | 0.1% |
| Glutamine | 0.0% | ± | 0.0% | 0.0% | ± | 0.0% | 1.2% | ± | 0.5% |
| Glycerol-3-phosphate | 0.0% | ± | 0.0% | 0.5% | ± | 0.2% | 0.9% | ± | 0.9% |
| UDP-glucose | 0.0% | ± | 0.0% | 0.1% | ± | 0.2% | 89.1% | ± | 9.9% |

Table S2: ^13^C dynamics in the hippocampal region dissected from 2-month-old male C57BL6 mice (n = 3 for each point)

| **EMP - Pathway** | 1m | | | 5m | | | 60m | | |
| --- | --- | --- | --- | --- | --- | --- | --- | --- | --- |
| Glucose | 25.2% | ± | 6.0% | 29.3% | ± | 0.6% | 63.7% | ± | 10.4% |
| Hexose-6-Phosphate | 0.0% | ± | 0.0% | 0.4% | ± | 0.4% | 11.1% | ± | 6.4% |
| Hexose-1,6-Phosphate | 0.4% | ± | 0.5% | 0.1% | ± | 0.2% | 19.1% | ± | 11.3% |
| Triose-phosphate | 0.0% | ± | 0.0% | 0.2% | ± | 0.0% | 9.8% | ± | 2.8% |
| Phospho-D-glycerate | 2.0% | ± | 1.0% | 14.1% | ± | 18.6% | 61.8% | ± | 16.7% |
| **Pentose phosphate pathway** |  |  |  |  |  |  |  |  |  |
| Ribose-5-P | 1.1% | ± | 1.0% | 2.7% | ± | 1.8% | 22.3% | ± | 9.0% |
| **TCA Cycle** |  |  |  |  |  |  |  |  |  |
| 2-Ketoglutaric acid | 0.1% | ± | 0.0% | 0.1% | ± | 0.0% | 0.1% | ± | 0.0% |
| Aconitic acid | 0.1% | ± | 0.1% | 0.2% | ± | 0.1% | 0.1% | ± | 0.1% |
| Citric acid | 3.4% | ± | 1.8% | 2.9% | ± | 0.7% | 78.1% | ± | 18.9% |
| Succinic acid | 0.0% | ± | 0.0% | 0.0% | ± | 0.0% | 0.7% | ± | 0.7% |
| **Other** |  |  |  |  |  |  |  |  |  |
| L-Glutamate | 0.0% | ± | 0.0% | 0.0% | ± | 0.0% | 11.8% | ± | 1.3% |
| D-Aspartic acid | 0.0% | ± | 0.0% | 0.0% | ± | 0.0% | 5.8% | ± | 0.5% |
| GABA | 0.0% | ± | 0.0% | 0.0% | ± | 0.0% | 0.0% | ± | 0.0% |
| Glutamine | 0.0% | ± | 0.0% | 0.0% | ± | 0.0% | 4.1% | ± | 0.6% |
| Glycerol-3-phosphate | 0.0% | ± | 0.0% | 0.9% | ± | 1.2% | 51.7% | ± | 8.2% |
| UDP-glucose | 0.0% | ± | 0.0% | 0.1% | ± | 0.2% | 94.5% | ± | 8.6% |

Table S3: ^13^C dynamics in the hippocampal region dissected from 2-month-old male C57BL6 mice in response to LTP induction (n = 3 for each point)


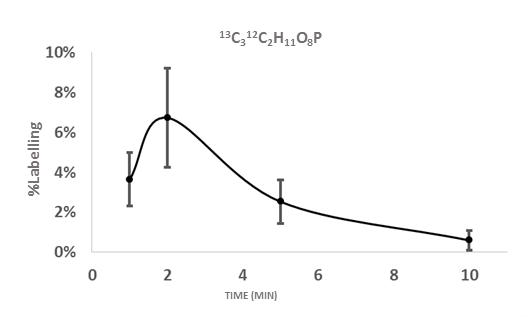


Figure S1: Dynamics of partially labelled pentose-5-phosphate with three labelled carbons (Formula ^13^C_3_^12^C_2_H_11_O_8_P; n = 4 for each point).


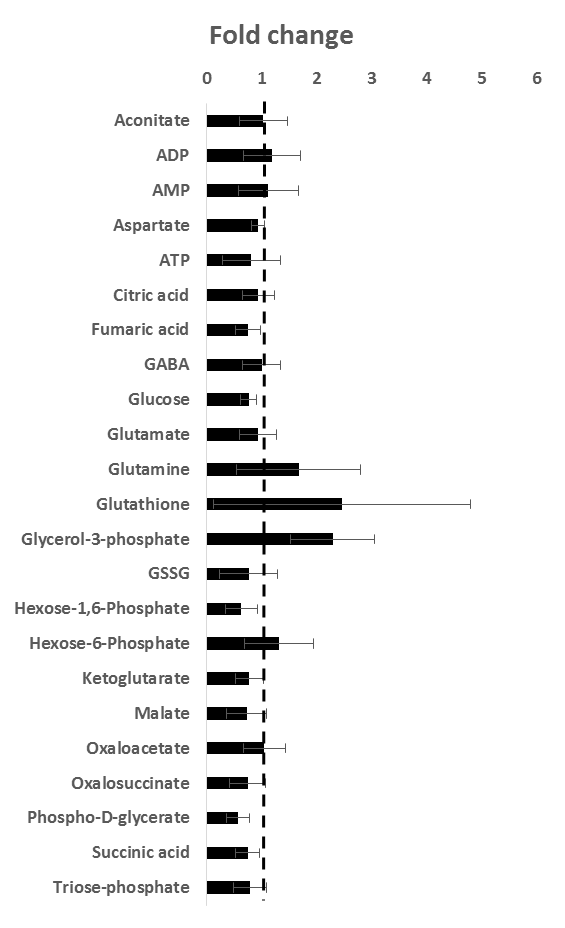


Figure S2: Fold change comparison between LTP (n = 3) and basal condition (n = 3) in hippocampal neurons from 2-month-old male C57BL6 mice.


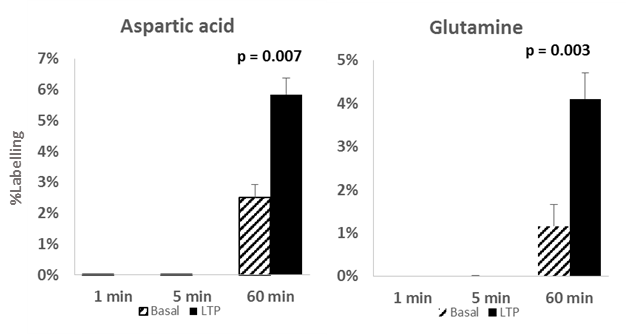


Figure S3: Dynamic labelling of aspartate and glutamine in basal conditions and after LTP induction. (n = 3 for each point).
